# Supplementary material for: Causal Relationship between Aging and Anorexia Nervosa: A White-Matter-Microstructure-Mediated Mendelian Randomization Analysis
Source: Biomedicines. 2024 Aug 16;12(8):1874. doi: 10.3390/biomedicines12081874 (PMC11351342; doi:10.3390/biomedicines12081874)
Supplement: Supplementary file 1 [file biomedicines-12-01874-s001.zip › Supplementary Figure/Figure S4/Singe SNP plot of MR analysis for TL to ubm-b-1971.pdf]

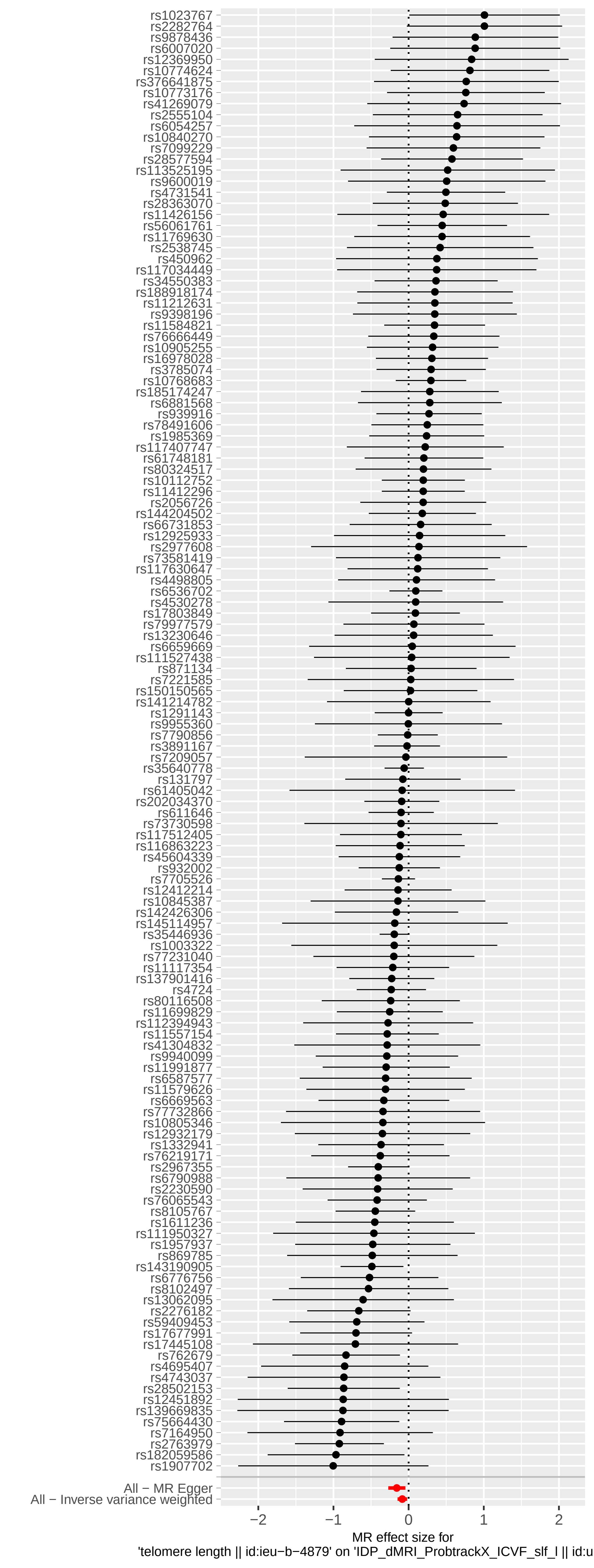
rs1023767
rs2282764
rs9878436
rs6007020
rs12369950
rs10774624
rs376641875
rs10773176
rs41269079
rs2555104
rs6054257
rs10840270
rs7099229
rs28577594
rs113525195
rs9600019
rs4731541
rs28363070
rs11426156
rs56061761
rs11769630
rs2538745
rs450962
rs117034449
rs34550383
rs188918174
rs11212631
rs9398196
rs11584821
rs76666449
rs10905255
rs16978028
rs3785074
rs10768683
rs185174247
rs6881568
rs939916
rs78491606
rs1985369
rs117407747
rs61748181
rs80324517
rs10112752
rs11412296
rs2056726
rs144204502
rs66731853
rs12925933
rs2977608
rs73581419
rs117630647
rs4498805
rs6536702
rs4530278
rs17803849
rs79977579
rs13230646
rs6659669
rs111527438
rs871134
rs7221585
rs150150565
rs141214782
rs1291143
rs9955360
rs7790856
rs3891167
rs7209057
rs35640778
rs131797
rs61405042
rs202034370
rs611646
rs73730598
rs117512405
rs116863223
rs45604339
rs932002
rs7705526
rs12412214
rs10845387
rs142426306
rs145114957
rs35446936
rs1003322
rs77231040
rs11117354
rs137901416
rs4724
rs80116508
rs11699829
rs112394943
rs11557154
rs41304832
rs9940099
rs11991877
rs6587577
rs11579626
rs6669563
rs77732866
rs10805346
rs12932179
rs1332941
rs76219171
rs2967355
rs6790988
rs2230590
rs76065543
rs8105767
rs1611236
rs111950327
rs1957937
rs869785
rs143190905
rs6776756
rs8102497
rs13062095
rs2276182
rs59409453
rs17677991
rs17445108
rs762679
rs4695407
rs4743037
rs28502153
rs12451892
rs139669835
rs75664430
rs7164950
rs2763979
rs182059586
rs1907702
All − MR Egger
All − Inverse variance weighted
−2
−1
0
1
2
MR effect size for
'telomere length || id:ieu−b−4879' on 'IDP_dMRI_ProbtrackX_ICVF_slf_l || id:u
